# Neural Activity When People Solve Verbal Problems with Insight

**DOI:** 10.1371/journal.pbio.0020097

**Published:** 2004-04-13

**Authors:** Mark Jung-Beeman, Edward M Bowden, Jason Haberman, Jennifer L Frymiare, Stella Arambel-Liu, Richard Greenblatt, Paul J Reber, John Kounios

**Affiliations:** **1**Department of Psychology, Northwestern UniversityEvanston, IllinoisUnited States of America; **2**Department of Psychology, Drexel UniversityPhiladelphia, PennsylvaniaUnited States of America; **3**Source Signal Imaging, IncSan Diego, CaliforniaUnited States of America

## Abstract

People sometimes solve problems with a unique process called insight, accompanied by an “Aha!” experience. It has long been unclear whether different cognitive and neural processes lead to insight versus noninsight solutions, or if solutions differ only in subsequent subjective feeling. Recent behavioral studies indicate distinct patterns of performance and suggest differential hemispheric involvement for insight and noninsight solutions. Subjects solved verbal problems, and after each correct solution indicated whether they solved with or without insight. We observed two objective neural correlates of insight. Functional magnetic resonance imaging (Experiment 1) revealed increased activity in the right hemisphere anterior superior temporal gyrus for insight relative to noninsight solutions. The same region was active during initial solving efforts. Scalp electroencephalogram recordings (Experiment 2) revealed a sudden burst of high-frequency (gamma-band) neural activity in the same area beginning 0.3 s prior to insight solutions. This right anterior temporal area is associated with making connections across distantly related information during comprehension. Although all problem solving relies on a largely shared cortical network, the sudden flash of insight occurs when solvers engage distinct neural and cognitive processes that allow them to see connections that previously eluded them.

## Abstract 2

Functional magnetic resonance imaging (fMRI) and electroencephalography (EEG) are used to study neural activity in subjects during a verbal task for which they report solutions achieved by insight

## Introduction

According to legend, Archimedes shouted “Eureka!” (“I have found it!”) when he suddenly discovered that water displacement could be used to calculate density. Since then, “Eureka!,” or “Aha!,” has often been used to express the feeling one gets when solving a problem with *insight*. Insight is pervasive in human (and possibly animal [Epstein et al. 1984]) cognition, occurring in perception, memory retrieval, language comprehension, problem solving, and various forms of practical, artistic, and scientific creativity (Sternberg and Davidson 1995). The Archimedes legend has persisted over two millennia in part because it illustrates some of the key ways in which insight solutions differ from solutions achieved through more straightforward problem solving. We examine the neural bases of these different problem-solving methods.

Although many processes are shared by most types of problem solving, insight solutions appear to differ from noninsight solutions in several important ways. The clearest defining characteristic of insight problem solving is the subjective “Aha!” or “Eureka!” experience that follows insight solutions (Schooler et al. 1993). This subjective experience can lead to a strong emotional response—according to legend, Archimedes ran home from the baths shouting “Eureka!” without donning his clothes first. In addition, problem solving with insight is characterized by the following features. (1) Solvers first come to an impasse, no longer progressing toward a solution (Duncker 1945). Archimedes, for example, was stymied by King Hiero's challenge to determine whether his new crown was pure gold without damaging the crown. (2) Solvers usually cannot report the processing that enables them to reinterpret the problem and overcome the impasse (Maier 1931). Insight often occurs when people are not even aware they are thinking of the problem, as reportedly happened to Archimedes while in the baths. (3) Solvers experience their solutions as arising suddenly (Metcalfe and Wiebe 1987; Smith and Kounios 1996) and immediately recognize the correctness of the solution (or solution path). (4) Performance on insight problems is associated with creative thinking and other cognitive abilities different from those associated with performance on noninsight problems (Schooler and Melcher 1997). Some researchers have argued that all these characteristics of insight solutions are essentially epiphenomenal, that insight and noninsight solutions vary only in emotional intensity, and that they are attained with precisely the same cognitive (hence neural) mechanisms (Weisberg and Alba 1981; Weisberg 1986; Perkins 2000).

Persistent questions about insight concern whether unconscious processing precedes reinterpretation and solution, whether distinct cognitive and neural mechanisms beyond a common problem-solving network are involved in insight, and whether the apparent suddenness of insight solutions reflects truly sudden changes in cognitive processing and neural activity.

Recent work suggests that people are thinking—at an unconscious level—about the solution prior to solving problems with insight. Specifically, while working on a verbal problem they have yet to solve, people presented with a potential solution word read the actual solution word faster than they read an unrelated word (Bowden and Beeman 1998). This “solution priming” effect is greater—and in fact people make solution decisions about presented words more quickly—when words are presented to the left visual hemifield, which projects directly to the right hemisphere (RH), than when words are presented to the right visual hemifield, which projects to the left hemisphere (LH). This suggests that RH semantic processing is more likely than LH semantic processing to produce lexical or semantic information that leads to the solution. These RH advantages occur only when solvers experience insight—the “Aha!” or “Eureka!” feeling that comes with insight solutions (Bowden and Jung-Beeman 2003a). Moreover, when subjects try to solve classic insight problems, they benefit more from hints presented to the left visual field (i.e., the RH) than from hints presented to the right visual field (i.e., the LH) (Fiore and Schooler 1998).

Problem solving is a complex behavior that requires a network of cortical areas for all types of solving strategies and solutions, so solving problems with and without insight likely invokes many shared cognitive processes and neural mechanisms. One critical cognitive process distinguishing insight solutions from noninsight solutions is that solving with insight requires solvers to recognize distant or novel semantic (or associative) relations; hence, insight-specific neural activity should reflect that process. The most likely area to contribute to this component of insight problem solving is the anterior superior temporal gyrus (aSTG) of the RH. Language comprehension studies demonstrate that the RH is particularly important for recognizing distant semantic relations (Chiarello et al. 1990; Beeman 1998), and bilateral aSTG is involved in semantic integration. For example, sentences and complex discourse increase neural activity in aSTG bilaterally (Mazoyer et al. 1993; Stowe et al. 1999), and discourse that places particular demands on recognizing or computing distant semantic relations specifically increases neural activity in RH temporal areas (St. George et al. 1999; Mason and Just 2004), especially aSTG (Meyer et al. 2000; Kircher et al. 2001). If this prediction of RH aSTG involvement is confirmed, it will help constrain neurocognitive theories of insight. Other cortical areas, such as prefrontal cortex and the anterior cingulate (AC) may also be differentially involved in producing insight and noninsight solutions.

We used functional magnetic resonance imaging (FMRI) in Experiment 1 and electroencephalogram (EEG) measurement in Experiment 2 to test the empirically and theoretically derived hypothesis that solving problems with insight requires engagement of (or increased emphasis on) distinct neural mechanisms, particularly in the RH anterior temporal lobe. Event-related experimental designs compared neural activity when people solved verbal problems with insight to neural activity when they solved problems (from the same problem set) without insight.

**Figure 1:**
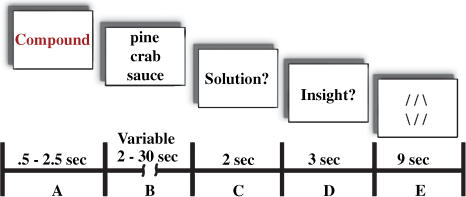
Sequence of Events for Each Trial (A) The “Compound” prompt was presented for 0.5 s, then persisted for a variable amount of additional time (0–2 s) until a cue from the scanner indicated the beginning of a new whole brain acquisition. (B) A three-word problem appeared in the center of the screen and persisted until subjects indicated with a bimanual button press that they had solved the problem, or until the 30-s time limit elapsed. Thus, event timing and condition were completely dependent on subjects' responses. (C) Following the button press or time limit, subjects were prompted to verbalize the solution (or press the buttons and say “Don't know” if the time limit expired prior to solution) then (D) prompted to indicate (with a bimanual button press) whether they felt insight, as described prior to the experiment. (E) Next, subjects performed 9 s of an unrelated filler task (three line-matching trials, 3 s each), allowing BOLD signal to return to baseline (in areas not involved in line matching).

As in earlier behavioral work, we used a set of compound remote associate problems (Bowden and Jung-Beeman 2003b) adapted from a test of creative cognition (Mednick 1962). Figure 1 illustrates the sequence for each trial. Subjects saw three problem words *(pine, crab, sauce)* and attempted to produce a single solution word *(apple)* that can form a familiar compound word or phrase with each of the three problem words *(pineapple, crab apple, applesauce)*. We relied on solvers' reports to sort solutions into insight solutions and noninsight solutions, avoiding the complication that presumed insight problems can sometimes be solved without insight (Davidson 1995) and circumventing the use of different types of problems requiring different cognitive operations. Thus, we made use of the most important defining characteristic of insight problems: the subjective conscious experience—the “Aha!” A similar technique revealed distinct behavioral characteristics when people recognized solutions with insight (Bowden and Jung-Beeman 2003a). Note that this is a very “tight” comparison. In both conditions problems are solved using a network of processes common to both insight and noninsight solutions. If insight ratings reflect some distinct cognitive processes, this contrast will reveal the distinct underlying brain activity. In other words, within the cortical network for problem solving, different components will be engaged or emphasized for insight versus noninsight solutions. FMRI (Experiment 1) should reveal neuroanatomical locations of processes that are unique to insight solutions, and EEG (Experiment 2) should reveal the time course (e.g., whether insight really is sudden) and frequency characteristics of neurophysiological differences.

## Results

### Experiment 1

Subjects solved 59% of the problems presented, and pressed buttons indicating “insight” for 56% (s.d. = 18.2) of their solutions, “no insight” for 41% (s.d. = 18.9) of their solutions, and “other” for 2% of their solutions. We marked a point about 2 s (rounded to the nearest whole second) prior to each solution button press as the solution event, and examined a time window 4–9 s after this event (i.e., 2–7 s after the button press) to isolate the corresponding hemodynamic response. Solving problems and responding to them required a strict sequence of events (reading of words, solving effort, solving, button press, verbalizing the solution, insight decision), but this sequence was identical whether subjects indicated solving with or without insight, so differences in FMRI signal resulted from the degree to which distinct cognitive processes and neural systems led to insight or noninsight solutions.

**Figure 2:**
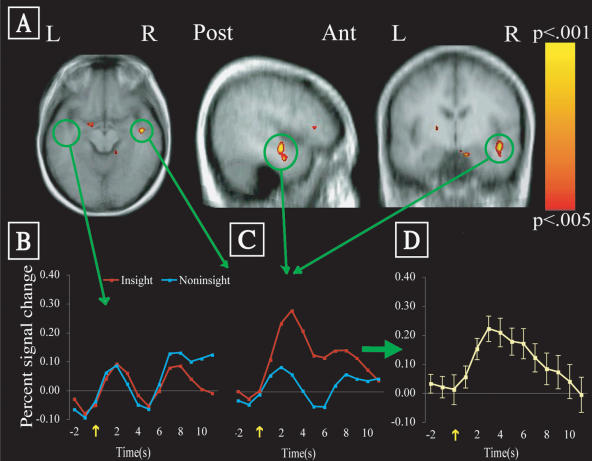
FMRI Insight Effect in RH aSTG (A) Voxels showing greater FMRI signal for insight than noninsight solutions, overlaid on the averaged normalized structural image of all subjects. The active area has a volume of 531 mm^3^ (peak *t* = 4.89 at 44, −9, −9 in Talairach space). (B) and (C) Group average signal change following the solution event, for insight (red line) and noninsight (blue line) solutions (yellow arrow indicates button press): (B) over entire LH aSTG region; (C) over entire RH aSTG region. (D) Insight solution signal change minus noninsight solution signal change, in RH aSTG (error bars show the standard error of the mean of the difference at each timepoint).

**Figure 3:**
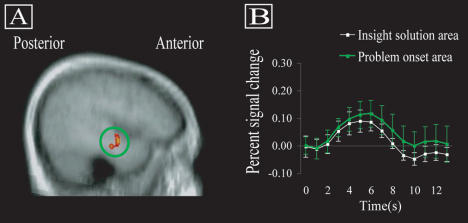
FMRI Signal in RH aSTG during Initial Solving Efforts (A) Voxels in right temporal lobe showing baseline-to-peak event-related FMRI signal when subjects first encounter problems, overlaid on the averaged normalized structural image of all subjects. The cluster is in RH aSTG, with a volume of 469 mm^3^, with peak *t* value of 4.37 at 41, −6, −12 in Talairach space, clearly overlapping with the cluster showing an insight effect at solution. (B) Group average signal change following problem onset (time = 0), for the cluster defined by signal at the problem onset (green line) and the cluster (illustrated in Figure 2A) showing the insight effect at solution (white line). Error bars show the standard error of the mean of the difference at each time point.

Figure 2 illustrates the most robust insight effect: as predicted, insight solutions were associated with greater neural activity in the RH aSTG than noninsight solutions. The active area was slightly anterior to primary auditory cortex, posterior to temporal pole, and along the medial aspect of the aSTG, extending down the lateral edge of the descending ramus of the Sylvian fissure to midway through the middle temporal gyrus (MTG). (This site is also close to the superior temporal sulcus, which has been implicated in language). Across all 13 subjects, the peak signal difference at a single voxel within the RH aSTG was 0.25% across the 6-s window, and 0.30% at a single time to repetition (TR), i.e., the time needed to repeat the image of the whole brain. Overall signal in this region was robust, reaching 96.8% of the brainwide average (after removing voxels in other brain areas with signal below a standard criterion). Within the cluster of voxels identified across the group, 12 subjects showed from 0.03% to 0.35% greater signal for insight than for noninsight solutions; one subject showed 0.02% greater signal for the noninsight solutions. It is not likely that RH aSTG is involved only in output or in emotional response following insight solutions, because neural activity in this area also increased when subjects first encountered each problem (Figure 3). Thus, RH aSTG is involved in processing the problem words both initially and at solution. (Of course, event-related FMRI signal occurred in many other cortical regions at problem onset, especially visual cortex). There was no insight effect in response windows immediately preceding or following the defined response window. All indications point to a striking transient event in the RH aSTG near the time when subjects solve problems with insight.

The involvement of the RH rather than the LH for this verbal task is not due to greater difficulty in producing insight solutions: subjects produced insight solutions at least as quickly (mean solution time = 10.25 s, s.d. = 3.58 s) as they produced noninsight solutions (mean = 11.28 s, s.d. = 4.13 s) (*t* < 1.0, *p* > 0.3). More importantly, the hemodynamic responses to both insight and noninsight solutions in the homologous area of the LH are about equivalent to the response to noninsight solutions in the RH aSTG—it is the strong response to insight solutions in the RH aSTG that stands out. There is no insight effect anywhere within temporal cortex of the LH. At statistical thresholds below significant levels (*p* < 0.1 uncorrected), there are as many voxels in LH temporal cortex showing a noninsight effect as showing an insight effect.

Several other cortical areas showing insight effects that did not meet significance criteria are listed in Table 1 (see also Figure S1). Some of these effects were in frontal cortex, which is notable because various frontal areas have been implicated in problem solving and reasoning. Patients with prefrontal damage have particular difficulty integrating relations in reasoning tasks (Waltz et al. 1999), and when healthy subjects perform the same task, neural activity increases in rostrolateral prefrontal cortext (Christoff et al. 2001). Some problem solving increases activity in dorsolateral prefrontal cortex (Prabhakaran et al. 1997), perhaps because of working memory demands. Solving of poorly structured problems seems particularly impaired following damage to the prefrontal cortex of the RH (Goel and Grafman 2000). Moreover, the inferior frontal gyrus (IFG) is highly active when people engage in directed semantic retrieval (Wagner et al. 2001) or when they select particular semantic concepts over competing ones (Thompson-Schill et al. 1997), e.g., to generate a response (Frith et al. 1991). Usually in these circumstances the IFG activity is stronger in the LH, even when people are reasoning about spatial problems (Goel et al. 1998), but the IFG responds particularly strongly in the RH when subjects select more distant semantic relations because of task demands (Seger et al. 2000) or comprehension goals (Robertson et al. 2000). Because of its putative importance for problem solving, semantic retrieval, and semantic selection, IFG was an a priori region of interest. One question we had hoped to answer was whether the semantic selection of insight solutions would preferentially evoke activity in RH or LH IFG, but the insight effects in both areas were too small (in area and in reliability) to test this question. When a more lenient statistical threshold was adopted, small clusters of signal were observed in both RH and LH IFG (Table 1; Figure S1A). Indeed, within the small region surpassing this weak statistical threshold, signal change in the RH IFG region was moderately strong (peak = 0.21% across the whole window). However, as is often the case, FMRI signal in this region was low (about 72% of the brainwide average) and variability was high, decreasing our confidence in the effect.

**Table 1:**
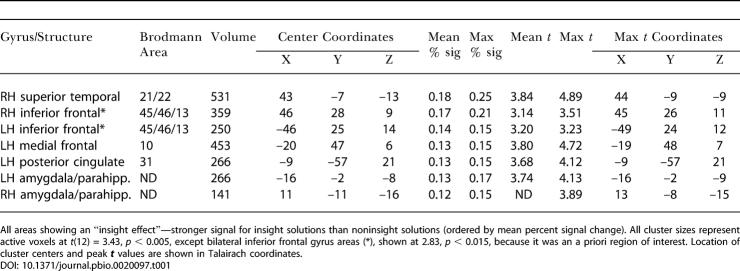
Full FMRI Results of Insight Effect

All areas showing an “insight effect”—stronger signal for insight solutions than noninsight solutions (ordered by mean percent signal change). All cluster sizes represent active voxels at *t*(12) = 3.43, *p* < 0.005, except bilateral inferior frontal gyrus areas (*), shown at 2.83, *p* < 0.015, because it was an a priori region of interest. Location of cluster centers and peak ***t*** values are shown in Talairach coordinates

After RH aSTG, the second largest area showing an insight effect in FMRI signal was the medial frontal gyrus in the LH (Table 1; Figure S1B). Although this area was 85% as large (453 mm^3^ at *p* < 0.005 threshold) as RH aSTG, the event-related signal within it was weak and the insight–noninsight difference (peak difference = 0.15%) was relatively small. (The insight effect may be attributable as much to a negative response for noninsight solutions as to a positive response for insight solutions.)

There also was an insight effect in small clusters in or near bilateral amygdala or parahippocampal gyrus. Again, regional signal was low (83% of the brainwide average), and the signal difference was small (peak = 0.16%). However, an amygdalar response may be expected, given the emotional sensation of the insight experience (Parsons and Osherson, 2001). Hippocampal or parahippocampal involvement is also plausible, if memory interacts with insight solutions differently from how it interacts with noninsight solutions. For instance, insight problems may encourage distinct memory encoding (Wills et al. 2000) or may require distinct retrieval. Finally, a small cluster in the LH posterior cingulate (PC) also showed an insight effect. There was strong, sustained FMRI signal for both solution types in this region; on the fringe of this responding region, FMRI signal began earlier following insight than noninsight solutions. The lateness of the FMRI signal across LH PC suggests that this effect began later in the response sequence, rather than during solution generation. Finally, as in most FMRI studies, signal was relatively weak in temporal pole and orbitofrontal areas due to magnetic susceptibility artifact, so we cannot rule out undetected effects in those areas.

Several cortical areas showed strong solution-related FMRI signal, but approximately equally for insight and noninsight solutions. Some of these areas (e.g., motor cortex) relate to the response sequence rather than solution processes; other areas probably reflect component processes of a problem-solving network common to both insight and noninsight solving, such as retrieving potential solutions. Two areas that may be of interest for future studies are AC and posterior middle/superior temporal gyrus. Both these areas, in the RH only, showed strong, negative solution-related signal, approximately equal in the two solution types. AC is an area that might be predicted to be involved in reorienting attention as solvers overcome impasses, given its role in performance monitoring and cognitive control (MacDonald et al. 2000). RH posterior MTG is active when subjects “get” jokes (Goel and Dolan 2001) and when they attempt to solve problems with deductive reasoning (Parsons and Osherson 2001). However, in our experiment, only the RH aSTG showed a robust insight effect.

### Experiment 2

A separate group of subjects participated in fundamentally the same paradigm while we continuously recorded EEGs from the scalp. We then compared time-frequency analyses of the EEGs associated with insight solutions versus noninsight solutions. EEG provides temporal resolution greatly superior to that of FMRI and thus can better elucidate the time course and suddenness of the insight effect. Furthermore, complex EEG oscillations can be parsed into constituent frequency components, some of which have been linked to particular types of neural and cognitive processes (Ward 2003).

The high temporal resolution of EEG allows us to address one of the fundamental questions raised earlier: does insight really occur suddenly, as subjective experience suggests? For problems typically solved without insight, solvers report gradually increasing closeness to solution. In contrast, for problems typically solved with insight, solvers report little or no progress until shortly before they actually solve the problem (Metcalfe 1986; Metcalfe and Wiebe 1987). Similarly, quantitative analyses of the distributions of response times and accuracies during anagram solving (a task frequently eliciting the experience of insight) reveal that a solution becomes available in a discrete transition from a state of little or no information about the correct response directly to the final state of high accuracy. This contrasts with various language and memory tasks not associated with insight, which yield partial outputs before processing has been completed (Kounios and Smith 1995; Smith and Kounios 1996).

We predicted that a sudden change in neural activity associated with insight solutions would produce an EEG correlate. Specifically, we predicted that high-frequency EEG oscillations in the gamma band (i.e., greater than 30 Hz) would reflect this sudden activity, because prior research has associated gamma-band activity with the activation of perceptual, lexical, and semantic representations (Tallon-Baudry and Bertrand 1999; Pulvermüller 2001). Gamma-band electrical activity correlates with the blood oxygenation level–dependent (BOLD) response apparent in FMRI signal; lower-frequency EEG components do not seem to have direct correlates in FMRI signal (Foucher et al. 2003; Laufs et al. 2003). Consequently, based on the language literature discussed earlier and on our FMRI results, we predicted a discrete insight-related increase in gamma-band activity at electrodes over the anterior temporal lobe of the RH.

Participants solved 46% (s.d. = 8.2) of the problems correctly within the time limit. Of correctly solved problems, subjects reported more insight solutions (56%, s.d. = 8.4) than noninsight solutions (42%, s.d. = 9.0), (*t*[18] = 3.47, *p*=0.003); there was no difference in mean response times (insight solutions = 9.94 s, s.d. = 2.60; noninsight solutions=9.25 s, s.d. = 3.06; t < 1.0).

**Figure 4:**
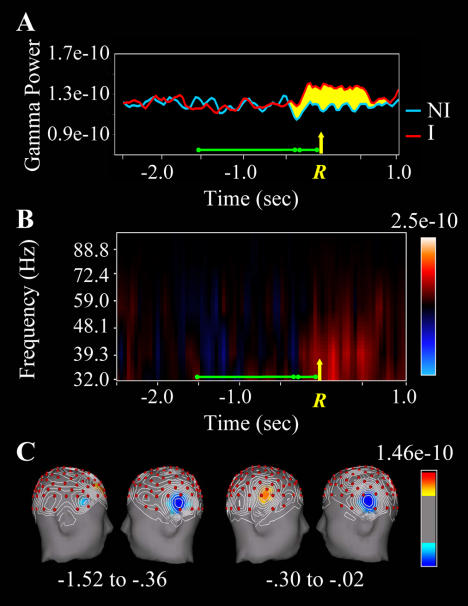
Gamma-Band Power for Insight and Noninsight Solutions (A) Grand average time course of EEG power (in v^2^) at 39 Hz estimated with the Morlet wavelet transform at right superior temporal electrode T8. The *x*-axis represents time (in seconds) with the yellow arrow and *R* marking the point in time of the solution button-press response (i.e., 0.0 s). The green horizontal bars above the *x*-axis represent the time intervals used in the statistical analyses and topographic maps. Note that gamma-band power for insight trials (red line) starts to increase above power on noninsight trials (blue line) by approximately 0.3 s before the button press. (B) Time-frequency plots of the insight minus noninsight difference shown in (A). The *y*-axis represents frequency (in Hz); the *x*-axis represents time (in seconds, with respect to the button press, exactly as shown in [A]). Red areas of the plot reflect times and frequencies at which insight EEG power is greater than noninsight EEG power; blue areas reflect times and frequencies at which noninsight EEG power is greater than insight EEG power. Note the sudden emergence of increased gamma power for insight solutions approximately 0.3 s before the button press. (C) Insight minus noninsight gamma-band differences plotted as topographic maps (LH and RH) of scalp current density (in v^2^/m^2^) estimated by a spline-based Laplacian transform computed with a realistic FMRI-derived head model. The Laplacian transform acts as a high-pass spatial filter that minimizes the contribution of activity distant from each electrode, thereby manifesting discrete, relatively superficial sources. The maps are thresholded to show foci of current density at the upper and lower 20% of the scale. Note the prominent effect of insight (effect for insight greater than effect for noninsight, in red) at the right superior temporal electrode (T8) and surrounding electrodes present from −0.30 to −0.02 s (measured with respect to the solution response) that is not present in the earlier epoch (−1.52 to −0.36 s). The blue area over left inferior parietal cortex (electrode P7) indicates that noninsight gamma power is nonsignificantly greater than insight power (*F*[1,19] < 1) over this region.

There was a burst of gamma-band activity associated with correct insight solutions (but not noninsight solutions) beginning approximately 0.3 s before the button-press solution response at anterior right temporal electrodes (Figure 4), with no significant difference between insight and noninsight solutions over homologous LH sites. A repeated-measures analysis of variance (ANOVA) performed on log-transformed gamma-band (39 Hz) EEG power at left and right temporal electrode sites (T7 and T8, respectively) for insight and noninsight trials using two time windows (−1.52 to −0.36 s and −0.30 to −0.02 s, measured with respect to the solution response) yielded significant insight × time window (*F*[1,18] = 6.68, *p* = 0.019) and insight × time window × Hemisphere (*F*[1,18] = 8.11, *p* = 0.011) interactions. The overall interaction occurred because there was an insight × hemisphere interaction from −0.30 to −0.02 s (*F*[1,18] = 4.61, *p* = 0.046) but no effect in the −1.52 to −0.36 s time window. Within the −0.30 to −0.02 s interval for these two electrodes, there was a significant insight effect at the right temporal (T8) site (*t*[18] = 3.48, *p* = 0.003), but not at the homologous left temporal (T7) site or any other LH temporal electrode. Laplacian mapping of this effect (Figure 4B) is remarkably consistent with the FMRI signal in RH aSTG observed in Experiment 1. (EEG does not have the spatial resolution of FMRI. However, we used the Laplacian transform [i.e., second spatial derivative] to localize observed activity. The Laplacian derivation acts as a high-pass spatial filter that reduces the contribution from activity in distant areas of the brain to the signal at a given electrode, and therefore reflects relatively focal and proximal brain activity. Given our FMRI results and the demonstrated correspondence between high-frequency EEG activity and FMRI signal [Foucher et al. 2003; Laufs et al. 2003], we are confident in the localization of this effect.)

The gamma burst in the right temporal area cannot be attributed to motor processes involved in making the response because (A) motor activity associated with the bimanual button press would have caused a bilateral gamma burst, not a unilateral one; (B) the location of the gamma burst as determined by Laplacian mapping (Figure 4B) is not consistent with hand-related motor cortex activity; and (C) both insight and noninsight solutions required button presses.

Other planned statistical tests (ANOVAs) examined possible insight-related frontal theta (5–8 Hz), posterior alpha (8–13 Hz), and fronto-central beta (13–20 Hz) activity. There were no statistically significant theta or beta effects. (Visual inspection and post hoc statistical tests suggested insight-related frontal 4-Hz activity, but this effect cannot be reliably distinguished from possible artifacts due to small vertical eye movements.) There was a significant posterior alpha effect, which is discussed below.

## Discussion

Complex problem solving requires a complex cortical network to encode the problem information, search memory for relevant information, evaluate this information, apply operators, and so forth. The FMRI and EEG results reported here conclusively demonstrate that solving verbal problems with insight requires at least one additional component to this cortical network, involving RH aSTG, that is less important to solving without insight. The insight effect in RH aSTG accords with the literature on integrating distant or novel semantic relations during language comprehension. When people comprehend (read or listen to) sentences or stories, neural activity increases in aSTG or temporal pole bilaterally more than when comprehending single words (Mazoyer et al. 1993; Bottini et al. 1994; Stowe et al. 1999; Humphries et al. 2001; Meyer et al. 2000). Neural activity increases in predominantly RH aSTG during tasks that emphasize integration across sentences to extract themes (St. George et al. 1999) or to form more coherent memories for stories (Mason and Just 2004). RH aSTG is also selectively active when subjects must generate the best ending to a sentence (Kircher et al. 2001) or mentally repair grammatically incorrect sentences (Meyer et al. 2000), both of which likely require intense semantic integration.

Like the results in language processing, the current results are predicted by the theory that the RH performs relatively coarse semantic coding (Beeman 1998; similarly, Chiarello et al. 1990). This theory contends that when people encounter words, semantic processing in several LH areas engages in relatively fine semantic coding which produces small semantic fields—i.e., this processing strongly focuses on a few concepts closely related to the input word in the given context. This is very effective for most straightforward language processing. In contrast, the homologous RH areas engage in relatively coarse semantic coding, which produces large and weak semantic fields—i.e., this processing includes many concepts, even concepts distantly related to the input words and context. This process is ineffective for rapid interpretation or selection but increases semantic overlap among multiple semantic fields (Beeman et al.1994), which is useful when drawing together parts of a story or conversation that are only distantly related (Beeman 1993; Beeman et al. 2000). In this view, the coarseness of semantic coding is largely influenced by slight asymmetries in neural microcircuitry that produce more discrete, less redundant input fields in pyramidal neurons of the LH language cortex, and more overlapping input fields in corresponding neurons in the RH (for reviews see Beeman 1998; Hutsler and Galuske 2003).

We suggest that semantic integration, generally, is important for connecting various problem elements together and connecting the problem to the solution, and that coarsely coded semantic integration, computed in RH aSTG, is especially critical to insight solutions, at least for verbal problems (or problems that can be solved with verbal or semantic information). People come to an impasse on insight problems because their retrieval efforts are misdirected by ambiguous information in the problem or by their usual method for solving similar problems. Large semantic fields allowing for more overlap among distantly related concepts (or distantly associated lexical items) may help overcome this impasse. Because this semantic processing is weak, it may remain unconscious, perhaps overshadowed by stronger processing of the misdirected information (Schooler et al. 1993; Smith 1995), and solvers remain stuck at impasse. Eventually, solution-related information bursts into awareness “in a sudden flash.” This can happen after misdirected processing decays or is suppressed, after solution-related processing grows, or after environmental cues occur—such as the water overflowing the bathtub when Archimedes got in. Archimedes had semantic and verbal knowledge about how to compute density from weight and volume, but struggled with measuring the volume of an irregularly shaped crown without harming the crown (e.g., melting it). His observation of water displacement allowed him to connect known concepts in new ways. This is the nature of many insights, the recognition of new connections across existing knowledge.

A persistent question has been whether the cognitive and neural events that lead to insight are as sudden as the subjective experience. The timing and frequency characteristics of the EEG results shed light on this question. We propose that the gamma-band insight effect in Experiment 2 reflects the sudden transition of solution-related cognitive processing from an unconscious to a conscious state. Recent research associates gamma-band oscillations with the ignition of neural cell assemblies supporting the transient feature binding necessary to activate a representation (Tallon-Baudry and Bertrand 1999; Pulvermüller 2001)—in this case, a phonological, lexical, or semantic representation corresponding to the solution word and its associations to the problem words. According to this hypothesis, greater synchronous gamma-band activity for insight than for noninsight solutions could reflect a more integrated or unitized solution representation. Furthermore, synchronous gamma-band activity has been hypothesized to play a critical role in the accessibility to consciousness of such representations (Engel and Singer 2001). The timing (with respect to the solution button press) of the insight gamma-band effect closely approximates estimates derived from cognitive behavioral studies of the amount of time required to access an available solution and generate a two-alternative, forced-choice button-press response (e.g., Kounios et al. 1987; Meyer et al. 1988; Smith and Kounios 1996). The present experiments had no response choice (i.e., always the same bimanual button press for solutions), so subjects could easily have responded 0.3 s after solving the problems. Thus, we infer that the observed gamma burst reflects the sudden conscious availability of a solution word resulting from an insight.

Suddenly recognizing new connections between problem elements is a hallmark of insight, but it is only one component of a large cortical network necessary for solving problems with insight, and recognizing new connections likely contributes to other tasks, such as understanding metaphors (Bottini et al. 1994) and deriving a story theme (St. George et al. 1999). Similar tasks may depend on related cortical networks. For example, appreciating semantic jokes (Goel and Dolan 2001) and engaging in deductive reasoning that sometimes involves insight (Parsons and Osherson 2001) both increase activity in RH posterior MTG. It is striking that the insight effect observed in the RH in our experiments occurred when people solved verbal problems, which traditional views suggest should involve mostly LH processing with little or no contribution from the RH. It is possible that insight solutions to nonverbal problems would require different cortical networks. However, the observed effect cannot be due simply to verbal retrieval, which must occur for both insight and noninsight solutions; it could be due to a type of verbal retrieval specific to insight solutions, but not involved in noninsight solutions.

**Figure 5:**
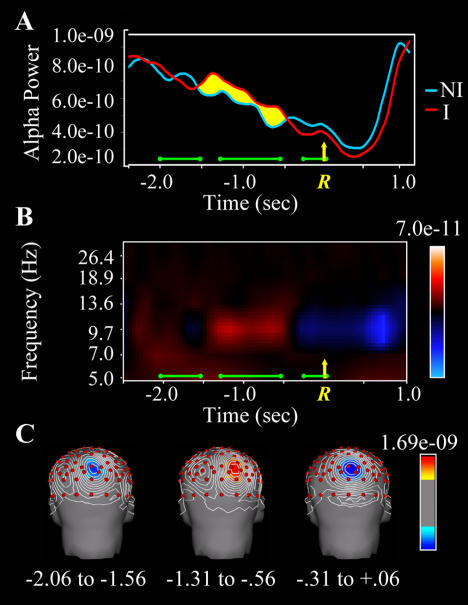
Alpha-Band Power for Insight and Noninsight Solutions (Same conventions as in Figure 4). (A) Time course of EEG power at 9.8 Hz (in v^2^) at right parietal-occipital electrode (PO8). The *x*-axis represents time (in seconds), with the green horizontal bars above the *x*-axis representing the time intervals used in the statistical analyses and topographic maps. The yellow arrow and *R* (at 0.0 s) signify the time of the button-press response. (B) Time-frequency plots of the insight minus noninsight difference shown in (A). (C) Insight minus noninsight alpha-band differences plotted as topographic maps of scalp current density (in v^2^/m^2^). Note that alpha-band power is significantly greater for insight solutions than noninsight solutions during the −1.31 to −0.56 s interval, but not during the preceding (−2.06 to −1.56 s) or subsequent (−0.31 to +0.06 s) intervals. This alpha burst was embedded in a slow decrease in alpha (see [A]), probably reflecting a general increase in cortical activity as effort increases during the course of problem solving.

We turn now to another result from the EEG time-frequency analysis, which was not predicted but nevertheless suggests a provocative interpretation. The gamma burst thought to reflect the transition of the insight solution from an unconscious to a conscious state was preceded by insight-specific activity in the alpha band (8–13 Hz). Specifically, there was a burst of alpha power (estimated at 9.8 Hz) associated with insight solutions detected over right posterior parietal cortex from approximately 1.4 s until approximately 0.4 s before the solution response, at which point insight alpha power decreased to the level of noninsight alpha power, or below (Figure 5). An ANOVA was performed on log-transformed alpha-band (9.8 Hz) EEG power at left and right parietal-occipital electrode sites (PO7 and PO8, respectively) for insight and noninsight trials using three time windows: −2.06 to −1.56 s, −1.31 to −0.56 s, and −0.31 to 0.06 s (measured from the solution button press). This analysis yielded a significant insight × time window interaction (*F*[2,36] = 4.13, *p* = 0.027, with the Huynh-Feldt correction). Follow-up *t*-tests in each time window yielded significant effects of insight in the first time window at both electrode sites (PO7: *t*[18] = 2.32, *p* = 0.033; PO8: *t*[18] = 2.42, *p* = 0.026) and in the second time window only at the RH site (PO8: *t*[18] = 2.17, *p* = 0.043), with a reversal of the direction of the effect. The third time window yielded no significant effects.

**Figure 6:**
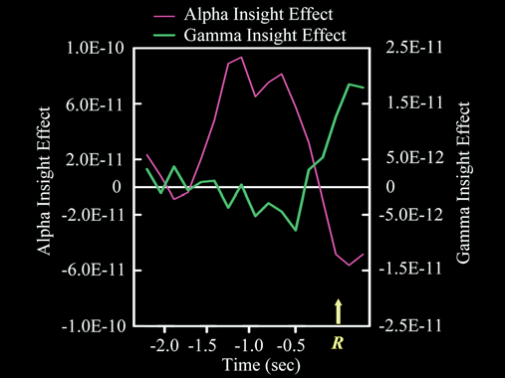
The Time Course of the Insight Effect Alpha power (9.8 Hz at right parietal-occipital electrode PO8) and gamma power (39 Hz at right temporal electrode T8) for the insight effect (i.e., correct insight solutions minus correct noninsight solutions, in v^2^). The left *y*-axis shows the magnitude of the alpha insight effect (purple line); the right *y*-axis applies to the gamma insight effect (green line). The *x*-axis represents time (in seconds). The yellow arrow and *R* (at 0.0 s) signify the time of the button-press response. Note the transient enhancement of alpha on insight trials (relative to noninsight trials) prior to the gamma burst.

Alpha rhythms are understood to reflect idling or inhibition of cortical areas (Pfurtscheller et al. 1996). Increased alpha power measured over parietal-occipital cortex indicates idling or inhibition of visual cortex. This has been attributed to gating of visual information flowing into the perceptual system in order to protect fragile or resource-intensive processes from interference from bottom-up stimulation (Ray and Cole 1985; Worden et al. 2001; Jensen et al. 2002; Cooper et al. 2003; Ward 2003). This interpretation assumes that brain areas are normally highly interactive, and that allowing one process to proceed relatively independently requires active attenuation of this interaction. For instance, when subjects attend to visual space in the hemifield projecting to one hemisphere, posterior alpha increases over the other hemisphere, which receives inputs from the unattended hemifield (Worden et al. 2001). Analogously, the present results suggest selective gating of visual inputs to the RH during the interval preceding the insight-related right temporal gamma burst (Figure 6). Hypothetically, this allows weaker processing about more distant associations between the problem words and potential solutions to gain strength, by attenuating bottom-up activation or other neural activity not related to solution that would decrease the signal-to-noise ratio for the actual solution.

This interpretation of the early insight-specific alpha effect is consistent with previous behavioral research suggesting that, prior to an insight, the solution to a verbal problem can be weakly activated (Bowers et al. 1990), especially in the RH (Bowden and Beeman 1998; Bowden and Jung-Beeman 2003a). Thus insight solutions may be associated with early unconscious solution-related processing, followed by a sudden transition to full awareness of the solution. We suggest that, in Experiment 2, the early posterior alpha insight effect is an indirect correlate of the former, and the right temporal gamma effect is a direct correlate of the latter.

In sum, when people solve problems with insight, leading to an “Aha!” experience, their solutions are accompanied by a striking increase in neural activity in RH aSTG. Thus, within the network of cortical areas required for problem solving, different components are engaged or emphasized when solving with versus without insight. We propose that the RH aSTG facilitates integration of information across distant lexical or semantic relations, allowing solvers to see connections that had previously eluded them. In the two millennia since Archimedes shouted “Eureka!,” it has seemed common knowledge that people sometimes solve problems—whether great scientific questions or trivial puzzles—by a seemingly distinct mechanism called insight. This mechanism involves suddenly seeing a problem in a new light, often without awareness of how that new light was switched on. We have demonstrated that insight solutions are indeed associated with a discrete, distinct pattern of neural activity, supporting unique cognitive processes.

## Materials and Methods

### 

#### Subjects

Ten men and eight women were paid to participate in Experiment 1; 19 new subjects (nine men, ten women) were paid to participate in Experiment 2. All were young (18–29) neurologically intact, right-handed, native English speakers; Experiment 1 participants met safety criteria for FMRI scanning. After hearing about all methods and risks and performing practice trials, they consented to participate. In Experiment 1, data from four men and one woman were excluded due to poor FMRI signal or because subjects provided fewer than ten insight or noninsight responses. This research was approved by the University of Pennsylvania Institutional Review Board.

#### Behavioral paradigm

Following practice, subjects attempted 124 compound remote associate problems during FMRI scanning. These problems (Bowden and Jung-Beeman 2003b) can be solved quickly and evoke an “Aha!” experience, producing a distinct behavioral signature (Bowden and Jung-Beeman 2003a), roughly half the time they are solved. Figure 1 illustrates the sequence of events for each trial. Each trial began with the task label “Compound” presented on liquid crystal diode goggles for 0.5 to 2.5 s. A gating signal from the scanner triggered the central presentation of three problem words, which persisted until subjects solved the problem or 30 s elapsed. If subjects solved the problem, they made a bimanual button press, after which the word “Solution?” prompted them to verbalize their solution. After 2 s the word “Insight?” prompted subjects to press buttons indicating whether they solved the problem with insight.

Prior to the experiment subjects were told the following: “A feeling of insight is a kind of ‘Aha!' characterized by suddenness and obviousness. You may not be sure how you came up with the answer, but are relatively confident that it is correct without having to mentally check it. It is as though the answer came into mind all at once—when you first thought of the word, you simply knew it was the answer. This feeling does not have to be overwhelming, but should resemble what was just described.” The experimenter interacted with subjects until this description was clear. This subjective rating could be used differently across subjects (or even across trials), blurring condition boundaries; yet the distinct neural correlates of insight observed across the group demonstrate that there was some consistency.

If subjects failed to solve problems within 30 s, the “Solution?” prompt appeared, and subjects pressed the “no” buttons and verbalized “Don't Know.” Then the “Insight?” prompt appeared, and subjects pressed the “no” buttons again. After the insight rating, subjects performed three line-matching trials (3 s each) to distract them from thinking about the problems, allowing the critical BOLD signal to return to baseline (Binder et al. 1999). The total time from the end of one problem to the onset of the next was 14.5–16.5 s. The condition (e.g., insight or noninsight solution) and time of events was determined by subjects' responses.

#### Image acquisition

Imaging was performed at the Hospital of the University of Pennsylvania, on a 1.5 Tesla GE SIGNA scanner with a fast gradient system for echo-planar imaging and a standard head coil. Head motion was restricted with plastic braces and foam padding. Anatomical high-resolution T1-weighted axial and sagittal images were acquired while subjects performed practice trials. Functional images (21 slices, 5 mm thick; 3.75-mm × 3.75-mm in-plane resolution; TR = 2000 ms for 21 slices; time to echo = 40 ms) were acquired in the same axial plane as the anatomical images using gradient-echo echo-planar sequences sensitive to BOLD signal (Kwong et al. 1992; Ogawa et al. 1992). Each functional run was preceded by a 20-s saturation period. Subjects participated in four 15-min runs and a fifth run of varying length, depending on the number of remaining problems.

#### Image analysis

Images were coregistered through time with a three-dimensional registration algorithm (Cox 1996). Echo planar imaging volumes were spatially smoothed using a 7.5-mm full-width half-maximum Gaussian kernel. Within each run, voxels were eliminated if the signal magnitude changed more than 10% across successive TRs, or if the mean signal level was below a noise threshold. Functional data were transformed (Collins et al. 1994) to a standard stereotaxic atlas (Talairach and Tournoux 1988) with a voxel size of 2.5 mm^3^.

Data were analyzed using general linear model analysis that extracted average responses to each trial type, correcting for linear drift and removing signal changes correlated with head motion. Each TR was divided into two 1-s images to improve time locking of the solving event and the functional image data (time-course data were temporally smoothed in Figures 2 and 3). Solution-related responses were calculated using the average signal change within the window 4–9 s (to account for hemodynamic delay) after the solving event (beginning about 2 s prior to the button press). Differences between insight and noninsight solution events were estimated for each participant, then combined in a second-stage random effects analysis to identify differences consistent across all subjects. A cluster threshold was set at regions at least 500 mm^3^ in volume (32 normalized voxels, or 7.1 original-sized voxels) in which each voxel was reliably different across subjects, (*t*[12] > 3.43, p < 0.005 uncorrected). Monte Carlo simulations with similar datasets reveal low false positive rates with these criteria. RH aSTG was the only cluster to exceed these criteria, and converging evidence and the a priori prediction about RH aSTG strengthen confidence in this result.

#### Experiment 2

Behavioral procedures were similar to those of Experiment 1, except that (A) problem words were presented at smaller visual angles to discourage eye movements, (B) there were 2-s delays between each event in the response sequence, and (C) subjects triggered a new problem directly after responding to the previous problem (i.e., no line task occurred between problems).

#### EEG methods

Continuous high-density EEGs were recorded at 250 Hz (bandpass: 0.2–100 Hz) from 128 tin electrodes embedded in an elastic cap (linked mastoid reference with forehead ground) placed according to the extended International 10–20 System. Prior to data analysis, EEG channels with excessive noise were replaced with interpolated data from neighboring channels. Eyeblink artifacts were removed from the EEG with an adaptive filter separately constructed for each subject using EMSE 5.0 (Source Signal Imaging Inc., San Diego, California, United States). Induced oscillations were analyzed by segmenting each subject's continuous EEG into 4-s segments beginning 3 s before each solution response. (An analysis epoch beginning at an earlier point in time would have resulted in the loss of trials associated with response times of less than 3 s.)

Time-frequency transforms (performed with EMSE 5.0) were obtained by the application of complex-valued Grossmann-Morlet wavelets, which are Gaussian in both time and frequency. Following Torrence and Campo (1998), the mother wavelet, ω_0_, in the time domain has the form



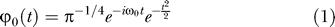



where ω_0_ is a nondimensional frequency. In this case, ω_0_ is chosen to be 5.336, so that ∫ϕ_0_(*t*) ≅ 0. The constant π−¼ is a normalization factor such that ∫(ϕ_0_(*t*))^2^ = 1. For the discrete time case, a family of wavelets may be obtained as



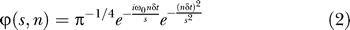



where δ*t* is the sample period (in seconds), *s* is the scale (in seconds), and *n* is an integer that counts the number of samples from the starting time. The Fourier wavelength λ is given by



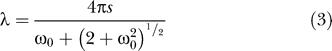



In the frequency domain, the (continuous) Fourier transform of Equation 2 is



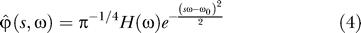



where



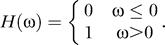



One reasonable way to measure the “resolution” of the wavelet transform is to consider the dispersion of the wavelets in both time and frequency. Since the wavelets are Gaussian in both domains, the *e*-folding time and frequency may serve as quantitative measures of dispersion. Note that these dispersions are a function of the scale, *s*. For a selected frequency, 𝒻_*c*_ = 1/λ, or from Equation 3



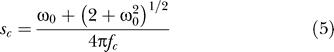



Then substituting into Equation 2, we find that the *e*-folding time is 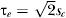 for frequency 𝒻_*c*_. From Equation 2, the *e*-folding frequency is 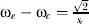. To make this concrete, we find that for a 10-Hz (alpha-band) center frequency, the *e*-folding time is 0.12 s and the *e*-folding frequency is 2.6 Hz. For a 40-Hz ( gamma-band) center frequency, the *e*-folding time is 0.03 s and the *e*-folding frequency is 10.5 Hz. Note that these *e*-folding parameters imply that wavelet scaling preserves the joint time-frequency resolution (equal areas in time-frequency space), with higher temporal resolution but broader frequency resolution as the wavelet scale decreases.

Segments corresponding to trials for which individual subjects produced the correct response were isolated and averaged separately according to whether or not the subject reported the experience of insight. Planned statistical tests (repeated-measure ANOVAs) were performed in order to detect insight-related effects on frontal midline theta (5–8 Hz), posterior alpha (8–13 Hz), fronto-central beta (13–20 Hz), and left and right temporal gamma (20–50 Hz). Response-locked event-related potentials (ERPs) were also computed using the same analysis epoch. Standard ERP analyses yielded no evidence of statistically significant effects, likely because ERPs reflect phase-locked activity rather than the induced (i.e., nonphase-locked) activity examined in the wavelet analyses; due to the long response times evident in this experiment, phase locking resulting from problem presentation would not be expected.

EEG effects were topographically mapped by employing spline-based Laplacian mapping with an FMRI-derived realistic head model and digitized electrode positions. Localization of EEG/ERP signals is a form of probabilistic modelling rather than direct neuroimaging. In contrast to other techniques, source estimation by Laplacian mapping indicates the presence of superficial foci of neuroelectric activity with minimal assumptions.

## Supporting Information

Figure S1 Cortical Regions Showing “Insight Effects” Below Cluster Size Threshold The far left lane shows for each region a single slice best depicting the cluster activated above threshold; middle lane shows time course of signal following insight (red line) and noninsight (blue line) solutions, across the entire active cluster; right panel shows the “insight effect” (insight signal minus noninsight signal, error bars show the standard error of the mean of the difference at each timepoint).
(A) depicts bilateral IFG with lowered threshold (*t*[12] = 2.83, *p* < 0.015); (B–D) depict clusters of FMRI signal at the same *t*-threshold used in the main paper (*t*[12] = 3.43, *p* < 0.005), but the clusters are too small to surpass cluster criterion.
(B) LH medial frontal gyrus;
(C) LH PC gyrus;
(D) LH amygdala (there was also a small cluster near RH amygdala). Spatial coordinates and other are details listed in Table 1.
(914 KB PDF).

## Abbreviations

AC: anterior cingulate
ANOVA: analysis of variance
aSTG: anterior superior temporal gyrus
BOLD: blood oxygenation level–dependent
EEG: electroencephalogram
ERP: event-related potential
FMRI: functional magnetic resonance imaging
IFG: inferior frontal gyrus
LH: left hemisphere
MTG: middle temporal gyrus
PC: posterior cingulate
RH: right hemisphere
TR: time to repetition.
