# Supplementary material for: Neural Activity When People Solve Verbal Problems with Insight
Source: PLoS Biol. 2004 Apr 13;2(4):e97. doi: 10.1371/journal.pbio.0020097 (PMC387268; doi:10.1371/journal.pbio.0020097)
Supplement: Figure S1 — The far left lane shows for each region a single slice best depicting the cluster activated above threshold; middle lane shows time course of signal following insight (red line) and noninsight (blue line) solutions, across the entire active cluster; right panel shows the “insight effect” (insight signal minus noninsight signal, error bars show the standard error of the mean of the difference at each timepoint). (A) depicts bilateral IFG with lowered threshold (t[12] = 2.83, p < 0.015); (B–D) depict clusters of FMRI signal at the same t-threshold used in the main paper (t[12] = 3.43, p < 0.005), but the clusters are too small to surpass cluster criterion. (B) LH medial frontal gyrus; (C) LH PC gyrus; (D) LH amygdala (there was also a small cluster near RH amygdala). Spatial coordinates and other are details listed in Table 1. (914 KB PDF). [file pbio.0020097.sg001.pdf]

Supplemental Figure legend

Supplementary Figure.

Cortical regions showing "insight effects" below cluster size threshold.

Far left panels show for each region a single slice best depicting the cluster activated above threshold; middle panel shows time course of signal following insight (red line) and noninsight (blue line) solutions, across the entire active cluster; right panel shows the "insight effect" (insight signal minus noninsight signal, error bars show SEM of the difference at each timepoint). (A) Bilateral IFG with lowered threshold ($t(12)=2.83$, $p<.015$); Sections (B-D) depict clusters of FMRI signal at the same t-threshold used in the main paper ($t(12)=3.43$, $p<.005$), but the clusters are too small to surpass cluster criterion. (B) LH medial frontal gyrus; (C) LH posterior cingulate gyrus; (D) LH amygdala (there was also a small cluster near RH amygdala). Spatial coordinates and other are details listed in Table 1.

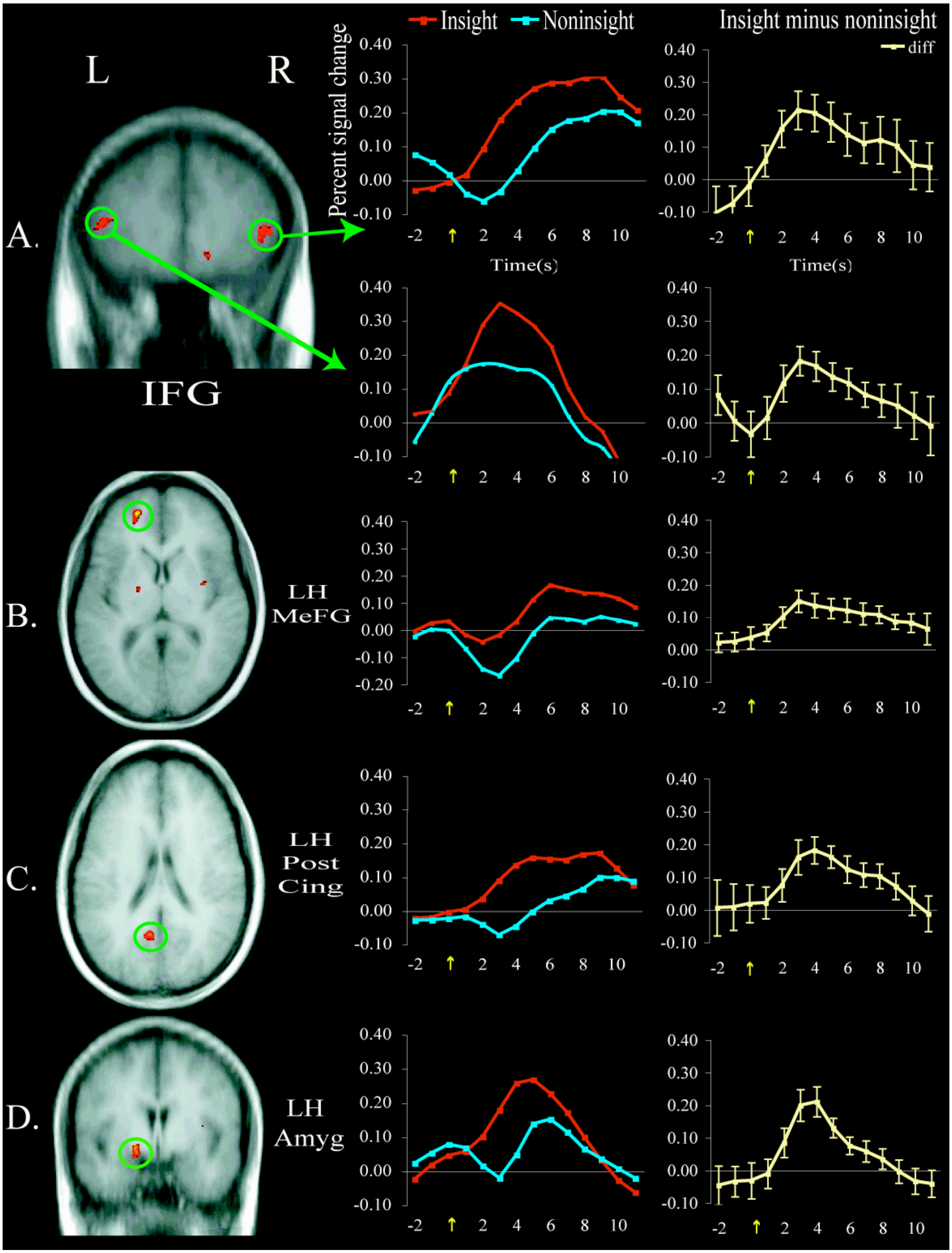
Insight
Noninsight
Insight minus noninsight
diff
Percent signal change
Time(s)
L
R
A.
IFG
B.
LH MeFG
C.
LH Post Cing
D.
LH Amyg
